# Searching for the Needles in a Haystack; Is It Needless? The Search for Peripheral Biomarkers in Psychiatry

**DOI:** 10.3389/fpsyt.2020.00689

**Published:** 2020-07-15

**Authors:** Maj Vinberg

**Affiliations:** ^1^Copenhagen University Hospital, Copenhagen, Denmark; ^2^Psychiatric Research Unit, Psychiatric Centre North Zealand, University Hospital of Copenhagen, Hillerød, Denmark

**Keywords:** biomarkers, severe mental illness (SMI), transdiagnostic, standard operating procedure (SOP), translation

## Introduction

Psychiatric diagnoses rely on syndrome description based on experienced symptoms as reported by patients and further transformed into diagnoses based on a professional’s (generally a doctor’s) education, knowledge and, not least, clinical experience. This is a major challenge and also an Achilles heel in psychiatry. The lack of more precise biological measures creates exposure to criticism against the use of psychiatric diagnoses: on the one hand, that psychiatric diagnoses are non-existent and, on the other hand, the challenge of demarking severe mental illness (SMI) from many minor psychiatric diagnoses that seem less biologically driven. Nevertheless, research methodologies such as proteomics, transcriptomics, genomics, and brain imaging have advanced the pathophysiological understanding of SMI (e.g. schizophrenia, bipolar disorder, and major depressive disorders) in particular (1–3). Although these efforts may seem promising, we face a translational gap in clarifying to what extent such approaches can prove useful in the clinic and help diagnostics to support a targeted treatment choice and optimize treatment overall. In particular, blood, plasma, and serum are untapped sources of possible clinical and research-useful biomarkers (1). Despite an increasing number of studies on biomarkers, so far the area has not contributed to solid clinically improvements in diagnostics or clinical care.

## Discussion

This paucity of progress is likely to be due to several factors that, potentially, could be overcome. First, our categorical diagnostic classification systems—the International Classification of Diseases, 10th revision (ICD-10), and the Diagnostic and Statistical Manual of Mental Disorders, 5th revision (DSM-V)—lump very heterogenic syndromes together based on empirical experience and these categories are not directed towards capturing the underlying biology. Consequently, most clinical research anchored in current diagnostic systems will, by nature, create narrow research questions such as: Do we believe that there are significant biological differences on comparing two severely depressed patients, one with unipolar disorder and one with bipolar disorder? One way to circumvent these limitations is to use initiatives such as the Research Domain Criteria (RDoC) framework (2), which has been created to integrate the observed behavioral information with neurobiological measures that incorporate multiple dimensions (behavior, thought patterns, neurobiological measures, and genetics), with the overall aim of generating a classification system that can be linked to the underlying dysfunctional pathways. Thus, future studies must look beyond the categorical diagnoses and use a transdiagnostic approach that includes a transdiagnostic assessment procedure for describing the individual behavior thoroughly. Along these lines, a systematic review (3) including the most promising peripheral biomarkers (BDNF, TNF-alpha, IL-6, C-reactive protein, and cortisol) concluded that these were most likely to be non-specific as diagnostic biomarkers (3). However, these biomarkers seem useful as an expression of illness activity and severity, thereby potentially being useful for treatment monitoring (3).

Second, the proteomic biomarker area must recognize that the earliest studies in particular are characterized by significant method limitations (4), such as low study quality (lack of consistency concerning the time of day when the biological samples are withdrawn; laboratory technique with a risk of prolonged storage before the biological samples have proceeded), poorly characterized samples, small sample sizes, and substantial unexplained between-study heterogeneity. Furthermore, limitations such as potential bias in individual studies, indications of publication bias, and lack of standard operating procedures for all aspects of the individual assessment can lead to non-replication and failed studies. These factors point to a need to improve the quality of future research. That said, knowledge from all these studies is essential because it can critically inform future warranted high-quality large-scale studies.

Third, it can be a problem to integrate animal models of SMI. However, close cooperation and infrastructures linking findings from animal models to clinical settings are beneficial (e.g. studying one biomarker in a mouse model and also observing the same potential biomarker in humans for both cases and controls). Integrating animal models is necessary to provide direct insight into the cellular metabolites that are produced during psychiatric processes. In addition, the influence on biomarkers due to short- or long-term medication can be observed under controlled conditions. Thus, a combination of representative animal models and human studies are a promising pathway to improve the potential use of biomarkers in psychiatry (5).

Fourth, there are huge commercial interests in this area. Companies are tempted to promise that their specially designed biomarker will capture the early signs of SMI and lead to better treatment results. This could be overly optimistic and will confuse the area, not least the patients. Thus, a combination of industry and academic groups that need to be funded can lead to being overly optimistic without a solid scientific basis for their promises should also be considered. Nevertheless, the impact of private companies’ knowledge, ambitious research, and technology is indispensable and well-described transparent cooperation between university-driven research and industry is necessary.

Fifth, another reason for the many non-replicated findings seems to be the inclusion of very different patient populations: it is difficult to compare biomarkers in a sample of newly diagnosed patients with patients having a late-stage disorder characterized by many admissions, polypharmacy, etc. Furthermore, the present illness state is important because biomarkers often change according to the present state (e.g. depressed, manic, psychotic, or in remission). Therefore, it is mandatory to characterize each illness state and illness course (onset of the disorder, number of episodes) as biomarkers change over time and are sensitive to the impact of environmental factors (smoking, substance use, alcohol use), co-existent physical disorder, age and gender; characterization of the menstrual cycle in females should also be taken into account.

Sixth, the impact of psychotropic medication on biomarker measures is complicated and a major confounder in most studies. This problem is difficult to solve but in larger studies it is possible to include at-risk individuals who do not receive medication (e.g. first-degree relatives to patients with SMI). Twin studies can further help to distinguish between potential environmental and genetic origins of the biomarker signatures (6).

Finally, measuring peripheral biomarkers at only one time point and using a case-control design is not an optimal design. Much more useful information is available if the trajectories of the biomarker are considered; for example, it may help to discriminate trait and state and whether a potential change can be replicated over time. Repeated observations over time including trajectory information are of major interest and also when using biomarkers as monitors of treatment response. Unfortunately, most studies on peripheral molecular biomarkers are cross-sectional studies using one time point to compare patients with controls (3). However, studying biomarkers at several time points makes it possible to evaluate their potential as state and treatment monitors and their use in the prediction of treatment response.

## Perspectives

Despite the above-described obstacles, overall there are, as described, promising peripheral biomarkers (3) that potentially will add to our future clinical tools and improve/impact clinical care. In particular, combining individual biomarkers across tissues and molecular systems seems to be a promising avenue for research in biomarker models (7, 8). However, instead of searching for the needles in the haystack, we need to collaborate and concentrate on describing all the elements in the haystack, integrating the inter-individual and intra-individual variability. At present, there is data and laboratory capacity and extended knowledge spread over multiple international research sites and industrial companies. However, creating well-structured multicenter studies is warranted, including mandatory structures for data sharing and integrated health information systems. Creating infrastructures and pipelines will move the field into the next stage of identifying pragmatically useful clinical biomarkers. This can be achieved by using innovative approaches and advanced technology (e.g. the R-LiNK initiative aimed at optimizing response to lithium treatment through personalized evaluation of individuals with bipolar I disorder) (9).

Evaluating the potential use of biomarkers or clusters of biomarkers as clinical tools in prevention, to assist in developing personalized medicine and in treatment monitoring, with an overall goal to facilitate more and better treatments for patients with SMI, is clearly needed. Researchers are encouraged to initiate a strategic research agenda (e.g. across a diagnostic biobank consortium) that will require the use of precise biomarker protocols (e.g. in line with the CONSORT 2010 checklist of information to include when reporting randomized trials) (10). Before initiation, a checklist stating a range of criteria, including gold standards, in all aspects using standardized operational procedures for all included studies, should be completed. This could also facilitate harmonization and joint databases, as in other areas of medicine (e.g. the Biomarker for Cardiovascular Risk Assessment across Europe consortium) (11). Overall, improving the opportunities for identifying the needles in the haystack.

## Author Contributions

The author confirms being the sole contributor of this work and has approved it for publication.

## Conflict of Interest

MV has received consultancy fees from Lundbeck, Sunovion and Janssen/Cilag in the past three years.
